# Enzymatic Synthesis of α-Glucosyl-Baicalin through Transglucosylation via Cyclodextrin Glucanotransferase in Water

**DOI:** 10.3390/molecules28093891

**Published:** 2023-05-05

**Authors:** Carole Lambert, Perrine Lemagnen, Eglantine Don Simoni, Jane Hubert, Alexis Kotland, Chantal Paulus, Audrey De Bizemont, Sylvie Bernard, Anne Humeau, Daniel Auriol, Romain Reynaud

**Affiliations:** 1 Givaudan France SAS, 31400 Toulouse, France; 2 NATEXPLORE SAS, 51140 Prouilly, France; 3 Givaudan France SAS, 22560 Pleumeur-Bodou, France

**Keywords:** baicalin, polyphenol, glucuronide, cyclodextrin glucanotransferase, α-cyclodextrin, enzymatic glucosylation, enzyme

## Abstract

Baicalin is a biologically active flavone glucuronide with poor water solubility that can be enhanced via glucosylation. In this study, the transglucosylation of baicalin was successfully achieved with CGTases from *Thermoanaerobacter* sp. and *Bacillus macerans* using α-cyclodextrin as a glucosyl donor. The synthesis of baicalin glucosides was optimized with CGTase from *Thermoanaerobacter* sp. Enzymatically modified baicalin derivatives were α-glucosylated with 1 to 17 glucose moieties. The two main glucosides were identified as Baicalein-7-*O*-α-D-Glucuronidyl-(1→4′)-*O*-α-D-Glucopyranoside (BG_1_) and Baicalein-7-*O*-α-D-Glucuronidyl-(1→4′)-*O*-α-D-Maltoside (BG_2_), thereby confirming recent findings reporting that glucuronyl groups are acceptors of this CGTase. Optimized conditions allowed for the attainment of yields above 85% (with a total glucoside content higher than 30 mM). BG_1_ and BG_2_ were purified via centrifugal partition chromatography after an enrichment through deglucosylation with amyloglucosidase. Transglucosylation increased the water solubility of BG_1_ by a factor of 188 in comparison to that of baicalin (molar concentrations), while the same value for BG_2_ was increased by a factor of 320. Finally, BG_1_ and BG_2_ were evaluated using antioxidant and anti-glycation assays. Both glucosides presented antioxidant and anti-glycation properties in the same order of magnitude as that of baicalin, thereby indicating their potential biological activity.

## 1. Introduction

**Figure 1:**
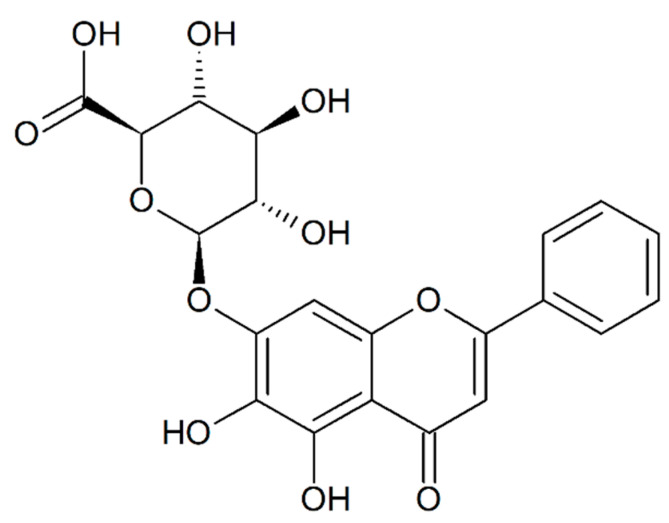
Chemical structure of baicalin (baicalein 7-*O*-glucuronide; 5,6-Dihydroxy-4-oxygen-2-phenyl-4H-1-benzopyran-7-β-D-glucopyranose acid).

Baicalin (baicalein-7-*O*-glucuronide, Figure 1) is found in *Scutellaria* sp. and more particularly in the perennial herb *Scutellaria baicalensis* Georgi. (Lamiaceae). Traditional Chinese medicine mentions the pharmacological properties of *S. baicalensis* roots as early as the first century B.C. [1]. The main bioactive constituents of these roots are flavonoids, including baicalin, baicalein, wogonoside, and wogonin, which are among the most representative. Baicalin and wogonoside are glucuronide forms of baicalein and wogonin, respectively. Substitution with glucuronide is less common than glucosylation in the plant kingdom, rendering baicalin a unique chemical structure (Figure 1).

Flavonoids generally display antioxidant and anti-inflammatory activities, and baicalin is not an exception. It presents reactive-oxygen-species-scavenging properties in human keratinocytes exposed to UV-B and UV-C, thereby decreasing cell apoptosis [2,3]. It also exerts anti-inflammatory properties, as described in a murine model of induced contact hypersensitivity [4]. In addition, baicalin induces the proliferation of human dermal papilla cells and inhibits the nuclear translocation of the androgen receptor stimulated by dihydrotestosterone in the same cells, suggesting its potential role as a cure for androgenetic alopecia [5]. 

Although baicalin is substituted with glucuronic acid, its water solubility is low, namely, 0.052 mg/mL (0.12 mM) [6], and thus limits its blending in water-based compositions. The use of cyclodextrins [7] or other formulation strategies [8], which can improve baicalin’s solubility by 5- to 50-fold but in restricted compositions, demonstrates the current research interest in finding new ways to increase baicalin’s hydrophilicity. Glucosylation is one approach to enhancing the water solubility of small molecules, and the use of an enzyme is particularly efficient compared to chemical routes that require multiple steps entailing protection/deprotection procedures, activated substrates, and catalysts and generally result in low yields [9]. Cyclodextrin glucanotransferases (EC.2.4.1.19) (CGTases), types of glucosyl transferase, catalyze the synthesis of cyclodextrins using starch and dextrin as donor substrate. Besides cyclization, CGTases also catalyze other reactions, such as coupling, which is the transfer of maltohexaose (opened α-cyclodextrin) to an acceptor; disproportionation, which is the transfer of glucose to maltopentaose to an acceptor; and the hydrolysis of maltohexaose [10]. In peculiar cases, CGTases more or less efficiently transglucosylate certain non-carbohydrate acceptors, including rutin, hesperidin, naringin, epigallocatechin gallate, stevioside, resveratrol, and ginseng saponins, which has been recently reviewed [10,11,12,13,14,15,16]. Natural glucosides are most conducive to the retrieval of good conversion yields, aside from two aglycones that had reported yields equal to or above 50%, namely, resveratrol and epigallocatechin gallate. Ngo et al. recently demonstrated that CGTase from *Thermoanaeroacter* sp. is able to transfer a glucosyl unit from α-cyclodextrin to octyl β-D-glucuronic acid on the C4 position of glucuronide [17], suggesting that baicalin could be an acceptor of this enzyme. 

This work aims to explore the potential of the transglycosylation of natural glucuronide. It presents an optimized process for the synthesis of baicalin glucosides using CGTase from *Thermoanaeroacter* sp. and *Bacillus macerans* in water along with a chemical characterization and preliminary physicochemical evaluation of baicalin mono- and diglucoside.

## 2. Results and Discussion

### 2.1. Comparison of Two CGTases

Baicalin glucosides were synthesized using CGTase Toruzyme^®^ 3.0L (Novozymes) from *Thermoanaerobacter* sp. and CGTase Amano from *Bacillus macerans*, for which α-cyclodextrin was employed as a glucosyl donor. The pH and temperature were set to 5.3 and 40 °C, respectively, to provide optimal conditions for CGTase Amano (the optimal conditions for Toruzyme^®^ are a pH of 5 to 5.5 and a temperature under 80–90 °C according to the manufacturer). The glucosyltransferase activity of the commercial preparations of CGTases Toruzyme^®^ and Amano was first measured on a model compound: salicin. The initial rates of salicin disappearance under the defined conditions were 30,131 and 22,441 µmol/min or units (U) per mL of commercial preparation for Toruzyme^®^ and CGTase Amano, respectively. The amount of enzyme to be employed for the glucosylation of baicalin was set at 0.37% (*v*/*v*), corresponding to 112 U/mL of the reaction medium. 

**Figure 2:**
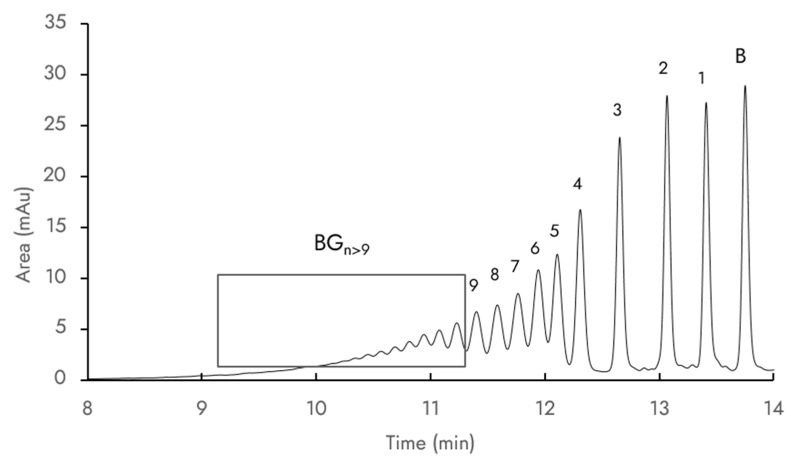
Example of an HPLC chromatogram at 280 nm obtained after transglucosylation of baicalin. Peaks: (B) baicalin, (1) baicalin-G_1_, (2) baicalin-G_2_, (3) baicalin-G_3_, (4) baicalin-G_4_, (5) baicalin-G_5_, (6) baicalin-G_6_, (7) baicalin-G_7_, (8) baicalin-G_8_, and (9) baicalin-G_9_. The grey square indicates a series of compounds that exhibits a UV spectrum (B) similar to baicalin and were termed BG_n>9_.

Figure 2 presents a typical UV chromatogram (280 nm) of the reaction mixture during transglucosylation. The chemical complexity of the mixture, which posed significant challenges in terms of chromatographic separation, prompted our use of a C8 HPLC column to improve the separation of mono-, di-, and triglucosides. Nine baicalin glucosides were clearly detected via LC/MS at a retention time ranging from 11.2 to 14 min. The molecular ions’ *m*/*z* values corresponding to each compound are summarized in Table 1. Other peaks with UV spectral absorption fingerprints similar to those of baicalin were observed from 9.0 to 11.2 min but their *m*/*z* values were not determined due to limitations of the simple quadrupole detector mass range parameters. It was hypothesized that these compounds are baicalin glucosides with more than nine glucose units and were thus labelled G_n>9_. Indeed, it has already been described that such high-molecular-weight glucosides are synthesized by CGTases: Gudiminchi et al. identified up to 12 glucose units grafted on ascorbic acid [18], and Akiyama et al. identified up to 32 glucose units on rutin [19]. This is explained by the ability of CGTase to perform disproportionation reactions leading to the extension of oligoglucosyl chains of baicalin glucosides. 

**Figure 3:**
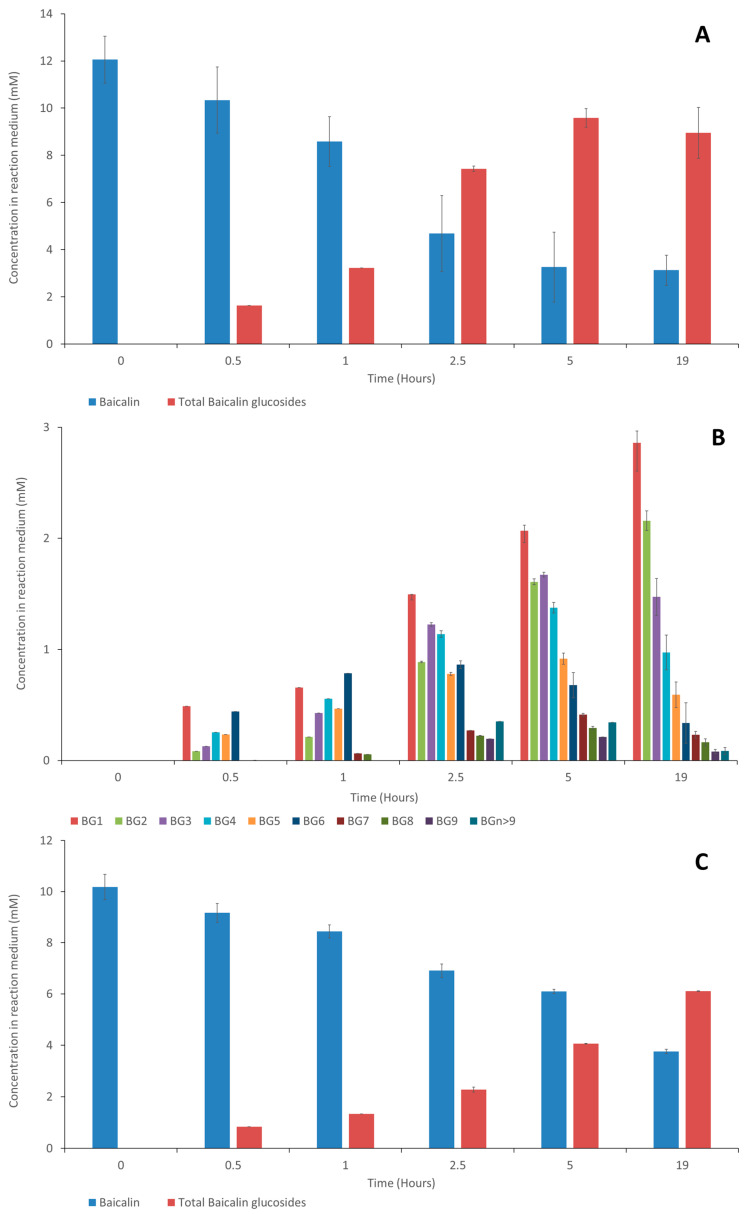

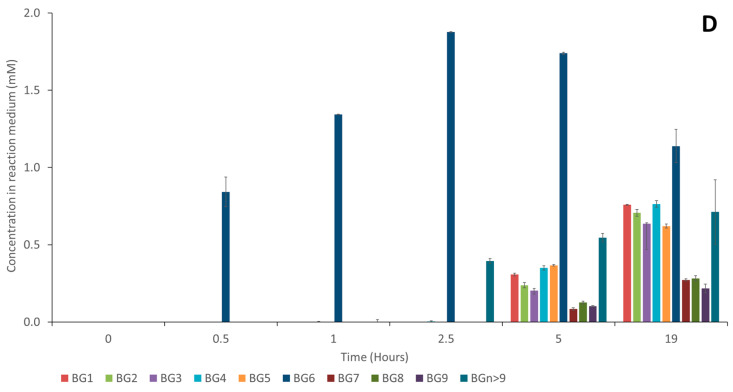
Time course of baicalin, total baicalin glucosides, and individual baicalin glucosides concentrations with Toruzyme*^®^* 3.0L (**A**,**B**) or with CGTase Amano (**C**,**D**) from 0 to 19 h of experiment. (Means of three replicates ± standard deviation.)

Figure 3 presents the time course of baicalin’s disappearance and baicalin glucosides’ appearance.

Regarding the synthesis of baicalin glucosides with Toruzyme^®^, BG_6_ was the main compound formed during the first hour, indicating that a coupling reaction was favored, followed by BG_1_ to BG_9_ in decreasing proportions. After 1 h, the concentration of BG_6_ decreased rapidly to the benefit of all the glucosides, showing that disproportionation, i.e., the transfer of maltopentaose and smaller glucose oligomers, became the major transglucosylation reaction, rapidly increasing the content of BGs 1–9. When using CGTase Amano, BG_6_ was also the first synthesized compound, and it remained the most dominant compound despite a slight decrease after 2.5 h and an increase in the concentrations of the other glucosides. This is in accordance with the research conducted by Rather et al. [20], who demonstrated that the CGTase Toruzyme^®^ is more prone to undergoing a disproportionation reaction than the CGTase Amano, leading to the highest concentrations of BG_1_ to BG_5_ being associated with Toruzyme^®^. At 19 h, the molar yield (total baicalin glucoside content/initial baicalin content) obtained with Toruzyme^®^ reached 79.5% of the total baicalin glucosides, and CGTase Amano afforded a 60% conversion. It is of great interest to note that both enzymes efficiently catalyze the transglucosylation of baicalin. This confirms the recent findings of Ngo et al. [17], namely, that glucuronide can be glucosylated by the CGTase from *Thermoanaerobacter* sp. In addition to the high yield of transglusylation afforded by Toruzyme^®^, the enzyme was more tolerant to pH variations than CGTase Amano in the range from 4 to 6 (Supplementary Data S1). Therefore, all other experiments were conducted with Toruzyme^®^.

### 2.2. Glucosylation of Baicalin, Baicalein, and Baicalein-7-O-Glucoside

Toruzyme^®^ transglucosylation efficacy varies widely depending on the chemical structure of the acceptor. Kaempferol, hesperetin, hydroquinone, resveratrol, ascorbic acid, and erythorbic acid have been reported to be transformed at yields lower than 30% [18,21,22,23,24,25], while naturally glucosylated molecules such as stevioside [15,26] and rutin [10,27] can be converted at up to 50–70%. This suggests that the native glucose unit increases enzyme/substrate affinity. The affinity of Toruzyme*^®^* for baicalin (baicalein 7-*O*-glucuronide), baicalein, and baicalein-7-*O*-glucoside was compared, for which the same conditions as previously described were maintained. Both the baicalin and baicalein-7-*O*-glucoside concentrations remained unchanged during the experiment and no other compounds were detected, suggesting that Toruzyme*^®^* cannot use it as acceptor. The results regarding baicalein-7-*O*-glucoside were particularly surprising, so the experiment was conducted again, yielding similar results. This renders the potential affordances of transglucosylate baicalin combined with CGTase even more interesting.

### 2.3. Optimization of Baicalin Glucoside Synthesis and Yield through Design of Experiment

The optimization of baicalin glucosides synthesis was achieved through an experiment and analysis via the response surface methodology. The variables (baicalin concentration, α-cyclodextrin concentration, temperature, enzyme content, and pH) and the data of the Central-Composite-Face-Centered (CCF) design applied are presented in Table 2. The limits of the variables were defined based on preliminary experiments that restricted the domain of optimization. A statistical analysis was used to discriminate the significance of the variables and their interactions. Multiple regressions were performed to provide an estimation of the yield and total content of baicalin glucosides. 

According to the F-value obtained via ANOVA (F = 38.8 for yield and F = 17.9 for total glucoside content, with 11 and 14 degrees of freedom, respectively) and the corresponding *p*-value < 0.001, both models were significant. No significant lack of fit was observed (*p* > 0.05; *p* = 0.35 for yield and 0.10 for total glucoside content). The correlation coefficients (R^2^ = 0.962 for yield and 0.947 for total glucoside content) indicated that 96% and 95% of the variability could be explained by the models established to describe baicalin glucoside yield and total baicalin glucoside content variation. 

**Figure 4:**
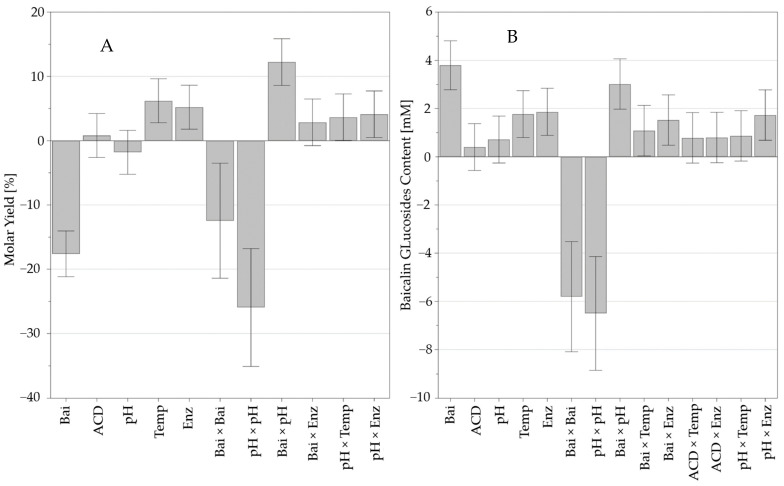
Regression coefficients of the quadratic models explaining the baicalin glucoside yield (**A**) and total baicalin glucoside content (**B**). Bai: baicalin concentration (mM), ACD: α-cyclodextrin concentration (mM), Temp: temperature (°C), and Enz: enzyme content (%, *v*/*v*). Variables positively impacting responses are in the upper part of the graph and vice versa with regard to those negatively impacting the responses. Significant results present error bars outside the 0 axis.

Figure 4 presents the coefficients and significance of the variables and interactions that led to the equations presented in Supplementary Data S2.

The most significant variable effects were baicalin concentration, the interaction between baicalin concentration and pH, and the quadratic effects of pH. The interaction of pH with the enzyme corresponded to a low level of significance. Moreover, previous work on the model phenolic compound, salicin (Supplementary Data S1), indicated that the transglucosylation capacity of Toruzyme^®^ was slightly variable within the pH range from 4 to 7. The acidic function of baicalin and its phenolic structure suggest different pKa values, and the molecular charge should vary depending on pH. This could explain the interaction between baicalin and pH and the non-linear effect of pH.

The temperature and enzyme amount also positively affected molar yield and total baicalin content. The improvement of the substrates’ solubility at higher temperatures and the ability of CGTase to function at 80 °C–90 °C (according to the manufacturer) could explain such positive impacts. 

**Figure 5:**
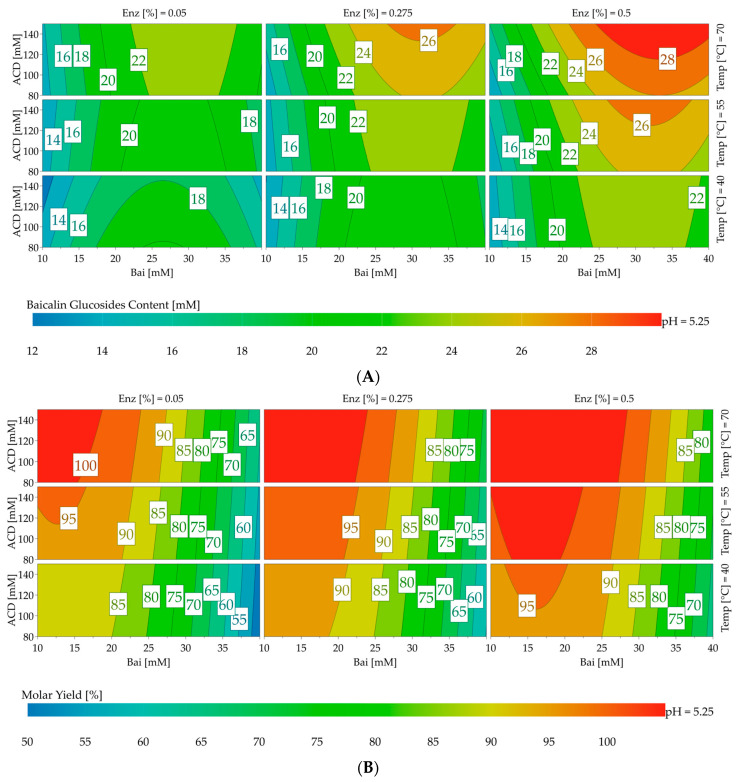
Response surface of central-composite-face-centered design of experiment for the optimization of baicalin glucoside content (**A**) and yield of the glucosylation reaction (**B**) at pH 5.25. The vertical left axis (ACD) corresponds to the α-cyclodextrin concentration (80–150 mM). The vertical right axis (Temp) corresponds to temperature (40–70 °C). Horizontal axis (Bai) corresponds to Baicalin concentration (10–40 mM). Columns from left to right describe contour plot for enzyme at 0.05% (15 U/mL), 0.275% (83 U/mL), and 0.5% (151 U/mL). Color scale from cold blue to hot red describes yield from 50 to 100% and the concentration of baicalin glucosides from 12 to 28 mM.

The response contour plots presented in Figure 5 allow for the visualization of the effects of the parameters, especially the initial concentration of baicalin, on both responses. The results were intentionally focused around pH 5.25, which offered the most interesting baicalin glucoside yields and content. 

Under the tested conditions, the maximum values for yield and total glucoside content were achieved using 0.5% CGTase Toruzyme*^®^* (151 U/mL) at 70 °C. The best yields, reaching up to 98.9%, were obtained in the 10–25 mM range of the initial baicalin concentration. Beyond 25 mM, the medium was more heterogenous and the yield decreased, but the total glucoside content increased and reached a maximum of 28.7 mM with baicalin at 40 mM and 150 mM of α-cyclodextrin. 

### 2.4. Synthesis, Purification, and Characterization of Baicalin Glucosides

**Figure 6:**
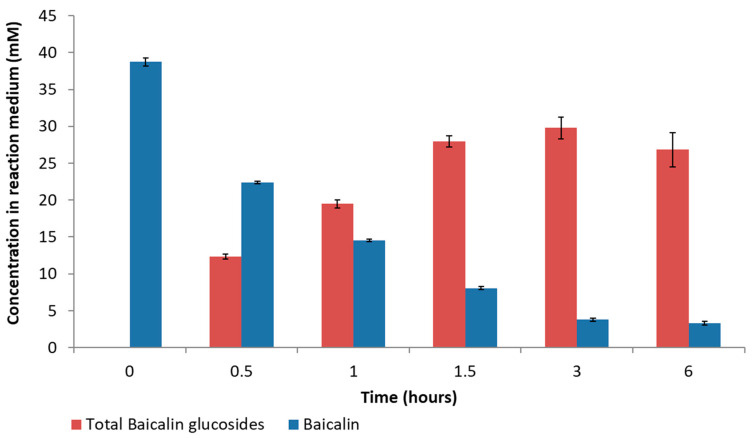
Time course evolution of baicalin and baicalin glucosides in the reaction medium. (Means of three replicates ± standard deviation.)

Baicalin glucosides were synthesized using the CGTase Toruzyme^®^ at a larger scale (500 mL) to better characterize the chemical diversity of the obtained mixture. Conditions were set according to the results obtained from the response surface methodology (see Figure 5A in the upper right corner) to maximize the content of total baicalin glucosides. The following parameters were employed: 38 mM of baicalin, 150 mM of α-cyclodextrin, pH 5.3, and an enzyme concentration of 0.5% (*v*/*v*, 151 U/mL). The glucosylation reaction was stabilized from 1 h up to 6 h (Figure 6), and the total glucoside content reached approximately 32.2 mM and a conversion yield of 84.6%. These values are aligned with those obtained by the established model, which predicted a 31.9 mM total glucoside value and a yield of 83.9%.

**Figure 7:**
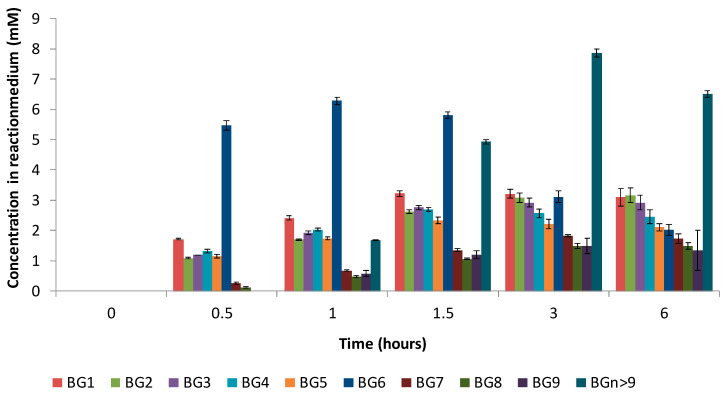
Time course evolution of individual baicalin α-glucoside in the reaction medium. (Means of three replicates ± standard deviation.)

Figure 7 shows the evolution of the different baicalin glucosides. Compared to the first synthesis performed with 20 mM of α-cyclodextrin (Figure 3), BG_1_ to BG_9_ followed a similar evolution, while the glucosides of higher molecular weights (termed BG_n>9_) were detected in higher concentrations, suggesting that a high concentration of α-cyclodextrin (150 mM) tends to favor the formation of these high-molecular-weight compounds. Indeed, increasing the α-cyclodextrin content could impact the rate of the disproportionation reaction and increase the probability of oligoglucosyl chain elongation.

The final reaction medium was purified to separate the carbohydrates produced from the hydrolysis of α-cyclodextrin and oligoglucosyl chains from the residual baicalin and its glucosides. To precisely characterize the chemical diversity of the baicalin glucosides, the whole mixture was analyzed via high-resolution LC/MS in the positive ion mode. In addition to the major mono- and diglucoside BG_1_ and BG_2_, LC/MS analyses confirmed the presence of the baicalin triglucoside to nonaglucoside and aided the elucidation of BG_n>9_; the baicalins decaglucoside to heptadecaglucoside were also detected in the purified reaction medium and annotated based on exact mass measurements (Table 3). The presence of heptadecaglucoside confirmed previous observations regarding the predilection of CGTase Toruzyme^®^ for disproportionation. The two major baicalin glucosides, BG1 and BG2, were separated from more complex glucosides to unambiguously determine their chemical structures via NMR.

**Figure 8:**
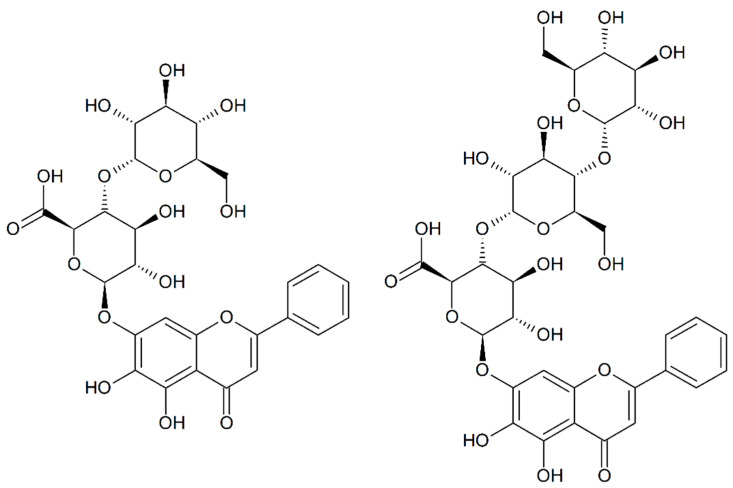
Chemical structures of baicalin monoglucoside (on the **left**) and baicalin diglucoside (on the **right**).

Centrifugal partition chromatography (CPC) was performed on the crude mixture of baicalin glucosides with a biphasic solvent system composed of methyl-*tert*-butyl-ether, *n*-butanol, and water in gradient elution mode. This first fractionation allowed us to concentrate on only one fraction baicalein, baicalin, baicalin monoglucoside, and baicalin diglucoside. This fraction was analyzed via 1D and 2D NMR, which showed that BG_1_ was Baicalein-7-*O*-β-D-Glucuronidyl-(1→4′)-O-α-D-Glucopyranoside (CAS 2548968-91-6) and that BG_2_ was Baicalein-7-*O*-β-D-Glucuronidyl-(1→4′)-*O*-α-D-Maltoside (Figure 8). The ^13^C and ^1^H NMR chemical shifts of BG_1_ and BG_2_ obtained from the 2D NMR experiments are presented in Supplementary Data S3 and S4.

To isolate BG_1_ and BG_2_ and evaluate their physicochemical properties, the purified reaction medium was subjected to deglucosylation with amyloglucosidase from *Aspergillus niger*. The deglucosylation process was optimized to increase the BG_1_ and BG*_2_* content without modifying the baicalin content. After deglucosylation, the concentrations of BG_1_ and BG_2_ increased by 3.6- and 3-fold, respectively, and a new CPC experiment allowed for the purification of BG_1_ and BG_2_ to 86.7% and 83.1% purity.

### 2.5. Evaluation of Baicalin Glucosides Properties

The solubility of BG_1_ and BG_2_ was compared to that of baicalin. The three compounds were solubilized in water. The solutions were centrifuged, and the compounds in the supernatant were quantified using HPLC. The water solubility of baicalin at 25 °C was 0.14 mM, in accordance with previous study (0.12 mM [6]). The water solubility of BG_1_ was 27.2 mM and that of BG_2_ was 46.3 mM, indicating improvements in water solubility by 188 and 320, respectively. The transglucosylation of baicalin allowed for a significant increase in its water solubility as compared to that obtained using formulation strategies [7,8]. 

The antioxidant and anti-glycation properties of the purified compounds BG_1_, BG_2_, and baicalin were evaluated. Reactive oxygen species (ROS) involved in the inflammatory process can lead to premature ageing of the skin, while antioxidant compounds can detoxify ROS and prevent inflammation. Glycation is a non-enzymatic chemical reaction that can take place in the heart of the dermis. Glucose molecules react with proteins, which leads to the disorganization of the dermis. Glycated proteins accumulate because they cannot be eliminated. Glucose binds the collagen and elastin fibers, which will stiffen and eventually break, leading to a loss of skin elasticity. That is why compounds that can inhibit the binding of carbohydrates to proteins are of interest for cosmetic applications.

Table 4 compares the half-maximal inhibitory concentration (IC50) of baicalin and its glucoside, which were measured during the antioxidant and anti-glycation assays.

The antioxidant activity of both glucosides was preserved, but was slightly lower than that of baicalin (1.5 to 1.8 times), and the IC50 values of the glucosides were lower than the reference (gallic acid, 0.059 mM). As the antioxidant activity of baicalin is related to the hydroxygroups of phenolic rings, it is not surprising to observe a slight modification of the antioxidant capacity of BG_1_ and BG_2_. Nevertheless, it is interesting to note that the glucosylation of phenolic hydroxyl groups does not always drastically reduce the antioxidant power of glucosylated compounds, as demonstrated with respect to epigallocatechin gallate [28]. The anti-glycation property of baicalin was more affected by glucosylation, leading to values that were 2 to 2.7 times lower for glucosides, but was once again lower than that of the positive control (aminoguanidine 0.36 mM). These results suggest that transglucosylation, which increased the water solubility of baicalin by 200- to 300-fold, slightly decreased the in tubo reactivity of baicalin. Further in vitro, ex vivo, and clinical studies are required to confirm the biological potential of baicalin glucosides. 

## 3. Materials and Methods

### 3.1. Chemicals

Baicalin, α-cyclodextrin, amyloglucosidase from *Aspergillus niger*, sulfuric acid (95%), 2,2-diphenyl 1-picrylhydrazyl, HPLC-grade acetonitrile, baicalein-7-*O*-glucoside, and acetic acid (99%) were purchased from Sigma Aldrich Chimie (Saint Quentin Fallavier, France). Cyclodextrin glucano-transferases were supplied by Univar (Genay, France) (Toruzyme*^®^* 3.0L) and Amano Enzyme Europe Ltd. (Milton, UK). Methyl tert-butyl ether and *n*-butanol were purchased from VWR International (Rosny sous bois, France).

### 3.2. HPLC Analysis of Baicalin and Baicalin-α-Glucosides

Quantification of baicalin and its α-glucosides was performed using an Ultimate 3000 system (Thermo Scientific, Courtaboeuf, France) composed of a quaternary pump (LPG 3400 SD), an autosampler (WPS 3000), and UV and MS detectors (DAD 3000 and ISQ EC, respectively). Software used was Chromeleon 7.2.10 ES. Column (Kinetex C8 2.6 µm, 4.6 × 100 mm Phenomenex, Le Pecq, France) temperature was maintained at 40 °C. Solvent A was ultrapure water with 0.1% acetic acid and solvent B was acetonitrile with 0.1% acetic acid. Flow rate was set at 1 mL/min, and LC gradient was as follows: 0 min, 10% B; 2 min, 10% B; 9 min, 20% B; 12 min, 45% B; 15 min, 45% B; 16 min, 10% B; and 20 min, 10% B. UV absorption was monitored at 280 nm. ISQ EC was in full-scan negative mode at 100–1000 Da, CID was 20 V, dwell scan time was 2 s, SIM widths were set at 0.10 amu, sheet gas pressure was 80 psi, auxiliary gas pressure was 9.7 psi, sweep gas pressure was 0.5 psi, and vaporizer temperature was 550 °C. Samples were prepared by diluting the reaction mixture twice with sulfuric acid at 0.5 M and then diluting it in a mix of acetonitrile/DMSO/water (2/2/6 *v*/*v*).

### 3.3. Comparison of Transglucosylation Capacity of Two CGTases toward Baicalin and Evaluation of Baicalein and Baicalein-7-0-Glucoside

Toruzyme*^®^* 3.0L was included in a transglucosylation experiment with baicalin, baicalein, or baicalein-7-*O*-glucoside at concentrations of 10 mM and α-cyclodextrin at 20 mM in an acetate buffer 60 mM at pH 5.3. The experiment was conducted for 19 h at 40 °C under agitation, and samples were collected regularly throughout the reaction. Reaction was stopped by adding 0.5 M of sulfuric acid, and samples were then diluted in acetonitrile/DMSO/water (2/2/6, *v*/*v*) before analysis using LC/MS.

### 3.4. Statistical Design of Experiment (DoE) for Baicalin Glucosylation Optimization

A Central-Composite-Face-Centered (CCF) design was applied to optimize the yield and the total glucoside content during the α-glucosylation of baicalin with CGTase. Modde^®^ Go 13.0.1 software (Sartorius, Aubagne, France) allowed us to design and analyze the CCF. Table 5 presents the variables and the levels employed, and Table 2 describes factors’ values for each experiment. CGTase volume is expressed in % (*v*/*v*) of commercial preparation in the reaction medium. The volume was set at 15 mL and duration at 16 h to ensure stabilization of the glucosylation reaction. Two responses were studied: total baicalin glucoside content (mM) and molar yield expressed as baicalin glucoside content/initial baicalin concentration. The following second-order polynomial equation was used to establish the relationship between variables and response:

$$Y=\alpha_{0}+\underset{i}{\sum }\alpha_{i}x_{i}+\underset{i}{\sum }{\alpha_{i}x_{i}}^{2}+\underset{ij}{\sum }\alpha_{ij}x_{i}x_{j}$$

where *Y* represents the response (molar yield or total glucoside content), $x_{i,\;}x_{j}$ denote the variables, $\alpha_{0}$ is a constant, and $\alpha_{i}$, $\alpha_{ij}$ are the linear, quadratic, and interaction coefficients. An analysis of variance (ANOVA) was performed to assess the significance of the correlations. 

### 3.5. Production, Fractionation, and Characterization of Baicalin α-Glucosides

The production of baicalin glucosides was conducted according to the optimal parameters defined via response surface methodology. Reaction was achieved with 38 mM of baicalin and 150 mM of α-cyclodextrin in 500 mL of sodium acetate buffer 60 mM at pH 5.3 at 70 °C. Reaction medium was sampled from 0 to 6 h and analyzed via LC/MS as previously described. After 6 h, the reaction was stopped by adding sulfuric acid (6 M) at up to pH 2.5. Reaction medium (555 g) was left to interact with adsorbent, macroporous resin (Diaion^®^ HP20, Sigma Aldrich) for 1 h. Resin was rinsed with water (2500 g) and eluted with 90% ethanol (1600 g) and water (360 g). Elution was concentrated under vacuum until reaching dryness. About 26 g of the baicalin α-glucoside mixture was obtained.

The baicalin α-glucoside mixture was analyzed via high-resolution LC/MS to tentatively identify the maximum number of baicalin glucosides, which was not easily elucidated via NMR solely due to peak overlaps. LC was conducted on Uptisphere C18 ODB 150 × 4.6 mm, 5 µm (Interchim). Mobile phase A was water with 0.1% formic acid and mobile phase B was acetonitrile with 0.1% formic acid. Flow rate was 0.7 mL/min and gradient was as follows: 0 min, 20% B; 0 to 17 min, 20–45% B; 17 to 18 min, 45 to 100% B; 18 to 24 min, 100% B; 24 to 25 min, 100% to 20% B; and 25 to 30 min, 20% B. Column oven was set at 35 °C, samples were maintained at 20 °C, and injection volume was 5 µL. Mass spectrometer used was SYNAPT G2 (Waters), and parameters were ESI+, *m*/*z* of 50–2000, and a scan rate of 1 s.

Baicalin α-glucosides were fractionated via centrifugal partition chromatography (CPC). CPC apparatus used was 250 PRO monitored via PLC 2250 (Gilson, Villiers-le-bel, France) with partition disks containing 240 cells of 0.9 mL. CPC experiment was employed to separate the baicalin residues and BG_1_ and BG_2_ from the other polyglucosylated forms of baicalin. Two biphasic solvent systems were prepared. System 1 was MTBE/*n*-butanol/water (4/1/5, *v*/*v*) and system 2 was MTBE/*n*-butanol/water (1/4/5, *v*/*v*). The column was filled with the lower phase of system 1 as the stationary phase. Approximately 1.7 g of the baicalin α-glucosides mixture was dissolved in 10 mL of lower phase and 5 mL of higher phase of system 1. After injection, elution was conducted with a gradient of the higher phase of system 1 and the higher phase of system 2 (at 20 mL/min and 1500 rpm and in ascending mode) as follows: from 0 to 10 min—100% higher phase system 1 and 0% higher phase system 2; from 10 to 80 min—higher phase of system 1 reached 0 and higher phase of system 2 reached 100%; from 80 to 110 min—0% higher phase system 1 and 100% higher phase system 2; and from 111 to 120 min the higher phase of system 2 was pumped in descending mode to proceed with extrusion. All fractions collected during the elution step were combined and evaporated under vacuum until dryness. An aliquot of this elution fraction (around 20 mg) was dissolved in 600 μL of DMSO-*d*6 and analyzed via ^13^C NMR at 298 K on a Bruker Avance AVIII-600 spectrometer (Karlsruhe, Germany) equipped with a cryoprobe. Elution fraction was also analyzed via 2D NMR experiments (HSQC, HMBC, and COSY).

### 3.6. Purification of Baicalin Mono- and Diglucoside for Testing

The baicalin α-glucosides mixture was subjected to amyloglucosidase treatment to increase the amount of baicalin monoglucoside (BG_1_) and baicalin diglucoside (BG_2_). Amyloglucosidase from *Aspergillus niger* was diluted 10 times in sodium acetate buffer (100 mM at pH 4.5). The reaction medium was composed of 6.2 g of α-glucosyl baicalin mixture, 1 mL of diluted amyloglucosidase, and 300 mL of sodium acetate buffer (100 mM at pH 4.5). The reaction was conducted at 55 °C for 15 min and stopped with sulfuric acid (6 M, ranging up to pH 2.5). Reaction medium (300 g) was left to interact with adsorbent resin for 1 h. Resin was rinsed with water (1140 g) and eluted with ethanol 90% (780 g). Elution was concentrated with a rotary evaporator until dryness. About 3.5 g of baicalin glucosides enriched in BG_1_ and BG_2_ was obtained.

A CPC experiment was conducted to purify BG_1_ and BG_2_. Two biphasic solvents were prepared as previously described in the first CPC experiment. System 1 was MTBE/*n*-butanol/water (4/1/5, *v*/*v*) and system 2 was MTBE/*n*-butanol/water (1/4/5, *v*/*v*). Column was filled with the lower phase of system 1 as stationary phase. Mixture enriched in BG_1_ and BG_2_ (0.51 g) was dissolved in 70% of the lower phase of system 1 and 30% of the higher phase of system 1. After injection, elution was conducted with a gradient of the higher phase of system 1 and the higher phase of system 2 (at 20 mL/min and 1200 rpm and in ascending mode) as follows: from 0 to 10 min—100% higher phase system 1, and 0% higher phase system 2; from 10 to 80 min—higher phase of system 1 reached 0 and higher phase of system 2 reached 100%; from 80 to 110 min—0% higher phase system 1, and 100% higher phase system 2; and from 111 to 120 min the higher phase of system 2 was pumped in descending mode. Fractions of 20 mL were collected. Based on UV monitoring at 280 nm and LC/MS analysis of fractions, fractions 41 to 56 (BG_1_) and 71 to 80 (BG_2_) were pooled and evaporated until dryness. The chemical structures of both compounds were confirmed via NMR as described above. 

### 3.7. Comparison of Solubility, Antioxidant Activity, and Anti-Glycation Activity of Baicalin and Its Glucosides

Solubility was determined at 25 °C using HPLC. Products were dissolved in water according to their solubility and were centrifuged. Then, the supernatant was diluted in acetonitrile/DMSO/water (2/2/6, *v*/*v*) to quantify the soluble portions of baicalin, BG_1_, and BG_2_. Baicalin was dissolved at 43.5 mg in 2 mL of water, BG_1_ at 114 mg in 5 mL of water, and BG_2_ at 64.1 mg in 1 mL of water.

The antioxidant activity of the three compounds was determined using the DPPH (2,2-diphenyl 1-picrylhydrazyl) free-radical-scavenging assay. The antioxidant activity was calculated by measuring the decrease in absorbance at 517 nm after incubation in the dark at room temperature for 120 min. The compounds were tested at different concentrations and diluted in water. DPPH solution was prepared in absolute ethanol at 0.0545 mg/mL. Each dilution of the molecule to be tested, each blank, and each positive control were deposited in triplicate in a 96-well microplate as follows: 75 μL of sample/gallic acid + 150 μL DPPH solution or 75 μL absolute ethanol + 150 μL DPPH (blank DPPH), or 75 μL sample + 150 μL absolute ethanol (blank sample). Gallic acid was used as positive control at a concentration of 1 to 7.52 µg/mL in ethanol. Calculation of DPPH reduction by baicalin and baicalin glucosides was determined as: % DPPH reduction = (1 − (Absorbance compound − Absorbance compound blank)/Abs DPPH blank) × 100. The IC50 corresponding to the concentration of the compound that reduced the DPPH by half was then calculated.

The anti-glycation properties of baicalin, BG_1_, and BG_2_ were also determined. The corresponding assays were performed in a 96-well micro-plate. A volume of 40 µL of each sample was mixed with 50 µL of BSA (bovine serum albumin, Sigma Aldrich) and 10 µL of ribose. The compounds were diluted in sodium phosphate buffer (0.1 M, pH 7.4). Several dilutions of the compounds were tested. A standard range of Aminoguanidine was used as inhibitor reference. Negative control contained sodium phosphate buffer and BSA. Positive control contained sodium phosphate buffer, BSA, and ribose.

Two blanks were developed for each compound: one with phosphate buffer, BSA, and sample, and one with phosphate buffer, ribose, and extract. The microplate was agitated and incubated for 17 h at 37 °C. Fluorescence was read with an excitation wavelength at 340 nm and an emission wavelength at 420 nm. Calculation of anti-glycation activity of extract was performed as follows: % inhibition of advanced glycation end-products’ formation = (1 − (IF extract − IF blank extract ribose or BSA)/(IF positive control-IF blank positive control) × 100. IF is intensity of fluorescence. The IC50, the concentration of the extract that inhibits advanced glycation end-product formation by half, was then determined.

## Figures and Tables

**Table 1:** Mass spectrometry data for identification of baicalin and its glucosides baicalin monoglucoside (BG_1)_ to baicalin nonglucoside (BG_9_).

Compound	Molecular Weight (g/mol)	Observed *m*/*z* MS^−^
Baicalin	446.4	445.0
BG_1_	608.5	607.0
BG_2_	770.6	768.9
BG_3_	932.8	931.2
BG_4_	1094.9	546.2
BG_5_	1257.1	627.7
BG_6_	1419.2	708.0
BG_7_	1581.4	789.3
BG_8_	1743.5	871.3
BG_9_	1905.6	951.8

**Table 2:** Central-Composite-Face-Centered design: value of variables (baicalin, α-cyclodextrin, pH, temperature, and enzyme concentration) for each experiment and corresponding responses (molar yield and glucoside content). Baicalin corresponds to the initial molar concentration of acceptor in the reaction medium. α-Cyclodextrin corresponds to the initial molar concentration of donor in the reaction medium. Enzyme corresponds to the amount of commercial preparation of CGTase Toruzyme initially added to the reaction medium: 0.5% is equivalent to 151 U/mL, 0.275% is equivalent to 83 U/mL, and 0.05% is equivalent to 15 U/mL. Experiments were conducted at the indicated pH and temperature for 16 h.

Baicalin	α-Cyclodextrin	pH	Temperature	Enzyme	Molar Yield	Glucoside Content
(mM)	(mM)		(°C)	(%, *v*/*v*)	(%)	(mM)
10	80	4.0	40	0.5	80.8	7.4
40	80	4.0	40	0.05	19	7.3
10	150	4.0	40	0.05	85.9	9.2
40	150	4.0	40	0.5	26.9	10.6
10	80	6.5	40	0.5	46.5	19.1
40	80	6.5	40	0.5	46.5	19.1
10	150	6.5	40	0.5	51.7	5.2
40	150	6.5	40	0.05	18.4	6.9
10	80	4.0	70	0.05	86.9	9.9
40	80	4.0	70	0.5	30.9	12.4
10	150	4.0	70	0.5	94	10.2
40	150	4.0	70	0.05	27.8	11.3
10	80	6.5	70	0.5	73.5	6.8
40	80	6.5	70	0.05	43.8	16.4
10	150	6.5	70	0.05	57.6	4.2
40	150	6.5	70	0.5	73.4	28.7
10	115	5.25	55	0.275	88.5	10.3
40	115	5.25	55	0.275	88.5	10.3
25	80	5.25	55	0.275	81.8	22.5
25	150	5.25	55	0.275	88.7	24.5
25	115	4.0	55	0.275	57.4	13.7
25	115	6.5	55	0.275	65.9	15.8
25	115	5.25	40	0.275	98.9	23.4
25	115	5.25	70	0.275	98.3	22.8
25	115	5.25	55	0.05	92.3	22.3
25	115	5.25	55	0.5	94.2	22.4
25	115	5.25	55	0.275	93.9	22.4
25	115	5.25	55	0.275	98.1	23.7
25	115	5.25	55	0.275	88.7	22.0

**Table 3:** High-resolution mass spectrometry data regarding the baicalins monoglucoside (BG_1_) to heptadecaglucoside (BG_17_).

Compound	Molecular Weight (g/mol)	Observed *m*/*z* MS^+^
BG_1_	608.1377	609.1458 [M + H^+^]
BG_2_	770.1905	771.1996 [M + H^+^]
BG_3_	932.2433	933.2508 [M + H^+^]
BG_4_	1094.2961	1095.3046 [M + H^+^]
BG_5_	1256.3489	1257.3574 [M + H^+^]
BG_6_	1418.4017	1419.4124 [M + H^+^]
BG_7_	1580.4545	1581.4619 [M + H^+^]
BG_8_	1742.5073	1743.5146 [M + H^+^]
BG_9_	1904.5601	1905.5641 [M + H^+^]
BG_10_	2066.6129	1045.3052 [M + H^+^ + Na]^2+^
BG_11_	2228.6657	1126.3312 [M + H^+^ + Na]^2+^
BG_12_	2390.7185	1207.8572 [M + H^+^ + Na]^2+^
BG_13_	2552.7713	1288.3823 [M + H^+^ + Na]^2+^
BG_14_	2714.8241	1369.4097 [M + H^+^ + Na]^2+^
BG_15_	2876.8769	1450.4386 [M + H + Na]^2+^
BG_16_	3038.9297	1531.4698 [M + H + Na]^2+^
BG_17_	3647.0674	1612.4863 [M + H + Na]^2+^

**Table 4:** Half-maximal inhibitory concentration (IC50) of baicalin, baicalin monoglucoside (BG1), and baicalin diglucoside (BG2) determined via anti-oxidant and anti-glycation tests.

Compound	IC50 Antioxidant Activity (mM)	IC50 Anti-Glycation Activity (mM)
Baicalin	0.025	0.044
BG_1_	0.037	0.122
BG_2_	0.045	0.091

**Table 5:** Levels of the different factors used to evaluate response surface methodology.

Variable (Unit)	Variable Coding		Levels	
		−1	0	+1
Temperature (°C)	Temp	40	55	70
CGTase (%, *v*/*v*)	Enz	0.05	0.275	0.5
α-cyclodextrin (mM)	Alp	80	115	150
Baicalin (mM)	Bai	10	25	40
pH	pH	4	5.25	6.5

## Data Availability

Data available on request due to privacy restrictions.

## Supplementary Materials

The following supporting information can be downloaded at: https://www.mdpi.com/article/10.3390/molecules28093891/s1, Supplementary Data S1: Influence of pH on CGTase Amano and Toruzyme*^®^* 3.0L transglucosylation activity using Salicin as substrate; Supplementary Data S2: Equations of models calculated using central-composite-face-centered design and explaining the molar yield of baicalin glucoside and the total baicalin glucoside content; Supplementary Data S3: ^1^H and ^13^C NMR chemical shifts of baicalin monoglucoside from NMR experiments in DMSO-*d6*; Supplementary Data S4: ^1^H and ^13^C NMR chemical shifts of baicalin diglucoside from NMR experiments in DMSO-*d6*.

## Sample Availability

Not available.
