# Supplementary material for: Enzymatic Synthesis of α-Glucosyl-Baicalin through Transglucosylation via Cyclodextrin Glucanotransferase in Water
Source: Molecules. 2023 May 5;28(9):3891. doi: 10.3390/molecules28093891 (PMC10180260; doi:10.3390/molecules28093891)
Supplement: Supplementary file 1 [file molecules-28-03891-s001.zip › molecules-2343241-supplementary.pdf]

molecules

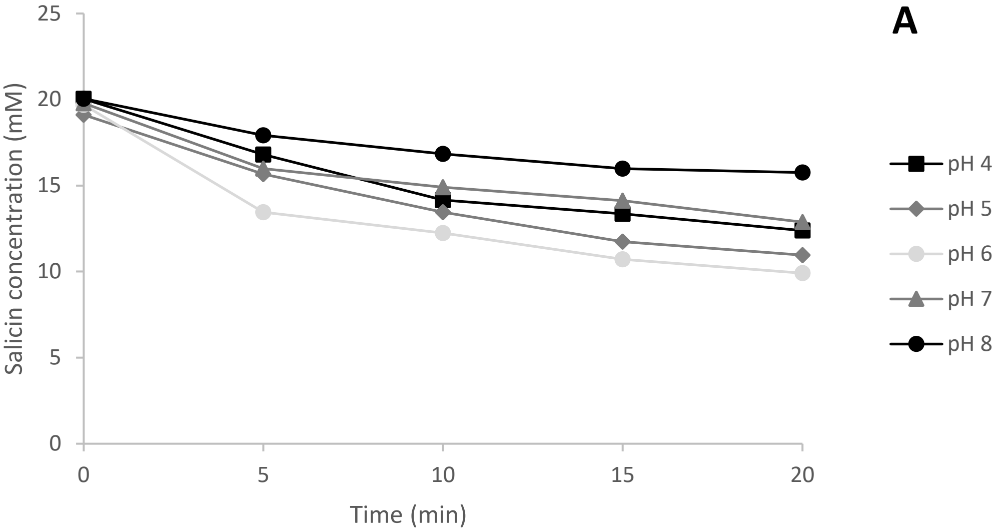

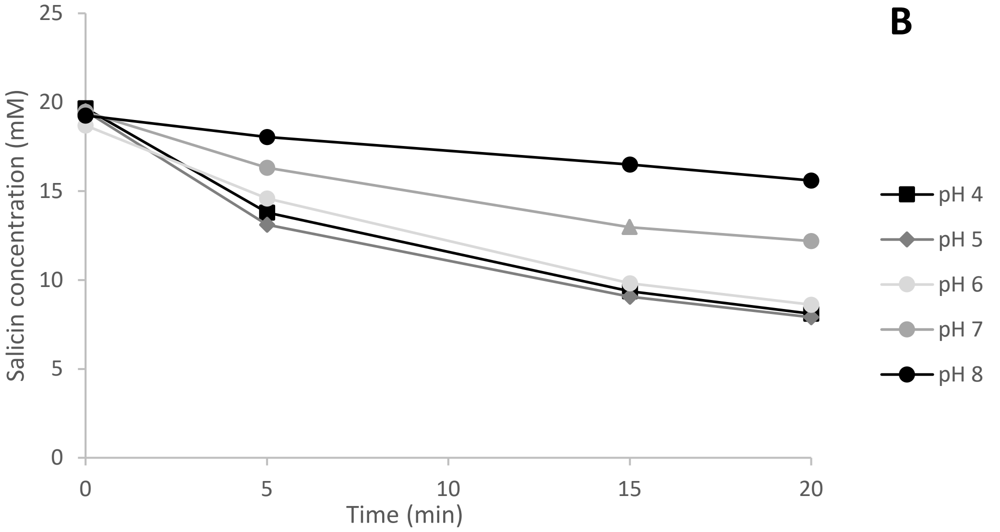

Supplementary Data S1. Influence of pH on CGTase Amano (**A**) and Toruzyme® 3.0L (**B**) transglucosylation activity using Salicin as substrate. Enzyme was 730 U/mL (w/w), salicin 20 mM, α-cyclodextrin 20 mM and experiment was conducted at 40°C from pH 4.0 to 8.0. Salicin disappearance is measured to follow enzyme activity. In the range of pH 4-7, Toruzyme® activity is very similar while it decreased at 8. CGTase Amano activity was similar at pH 5-7 but decreased significantly outside this range.

(1) Baicalin glucosides Yield (%) = 92.65 -15.79 x Bai + 1.78 x CD - 2.76 x pH +7.17 x Temp + 6.17 x Enz - 9.3 x Bai^2 - 30.49 x pH^2 +13.3 x Bai x pH + 1.76 x Bai x Enz + 4.73 x Temp x pH + 5.2 x Enz x pH

(2) Total glucosides content (mM) = 23.01 + 4.25 Bai + 0.35 CD + 0.77 x pH +1.7 x Temp + 1.8 x Enz – 3.55 x Bai^2 - 8.85 x pH^2 + 2.95 x Bai x pH^2 +1.14 x Bai x pH

Supplementary Data S2. Equations of models calculated using centered Central-Composite Face-Centered design and explaining the molar yield (1) of baicalin glucoside and the total baicalin glucoside content (2).

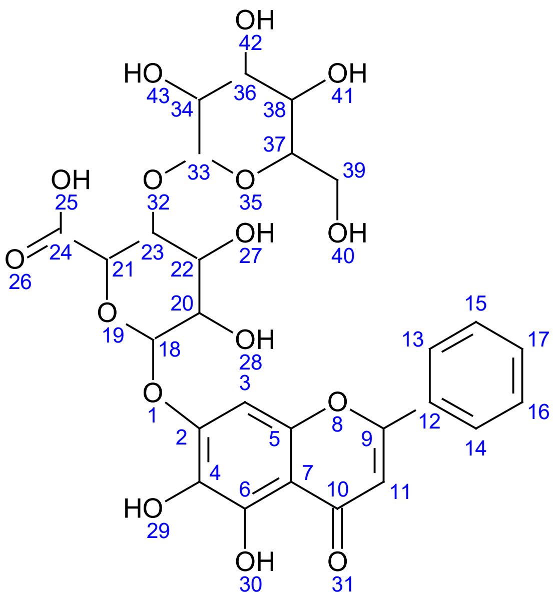

Atom number	δ ^{13}C (ppm)	δ ^{1}H (ppm)
2	151.6	-
3	93.9	7.04
4	131.2	-
5	149.6	-
6	147.1	-
7	106.7	-
9	163.8	-
10	182.8	-
11	104.9	7.02
12	132.5	-
13,14	126.5	8.07
15,16	129.3	7.61
17	132.2	7.62
18	99.7	5.33
20	72.4	3.50
21	74.3	4.27
22	75.6	3.65
23	80.0	3.66
24	169.7	-
33	100.8	5.10
34	72.8	3.24
36	73.7	3.39
37	73.2	3.44
38	69.2	3.23
39	73.2	3.44

Supplementary Data S3. ^{1}H and ^{13}C chemical shifts of baicalin monoglucoside from NMR experiments in DMSO-*d6*.

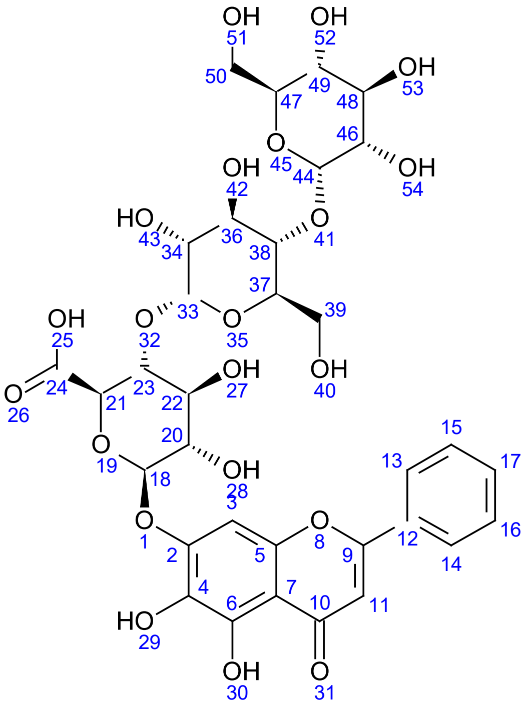

Atom number	δ ^{13}C (ppm)	δ ^{1}H (ppm)
2	151.8	-
3	94.4	6.99
4	131.5	-
5	149.5	-
6	147.2	-
7	106.7	-
9	164.0	-
10	183.1	-
11	105.2	6.99
12	131.3	-
13,14	126.8	8.07
15,16	129.7	7.6
17	132.6	7.61
18	100.5	5.23
20	72.6	3.48
21	75.5	4.16
22	75.1	3.68
23	80.6	3.70
24	170.7	-
33	99.7	5.14
34	72.3	3.28
36	73.6	3.65
37	71.6	3.65
38	79.7	3.36
39	60.5	3.62
44	101.2	5.00
46	73.0	3.23

47	73.9	3.49
48	73.9	3.40
49	70.4	3.07
50	61.2	3.62

Supplementary Data S4. ^{1}H and ^{13}C chemical shifts of baicalin diglucoside from NMR experiments in DMSO-*d6*.
